# Superb microvascular imaging ultrasound of the knee in patients with juvenile idiopathic arthritis—a repeatability study

**DOI:** 10.3389/fped.2025.1759370

**Published:** 2026-01-26

**Authors:** Martha Dohna, Faekah Gohar, Markus Neuhäuser, Doris Franke, Nima Memaran, Anna Raab, Jens Drube, Frank Dressler, Daniel Windschall

**Affiliations:** 1 Clinic for Diagnostic and Interventional Radiology, Department of Pediatric Radiology, University Hospital Bonn, Bonn, Germany; 2 Institute for Diagnostic and Interventional Radiology, Hannover Medical School, Hannover, Germany; 3 Department of Pediatric Rheumatology, St. Josef-Stift Sendenhorst, Sendenhorst, Germany; 4 Institute for Medical Biometry, Informatics and Epidemiology, University Hospital Bonn, Bonn, Germany; 5 Department of Pediatric Kidney, Liver, Metabolic and Neurological Diseases, Hannover Medical School, Hannover, Germany; 6 Department of Pediatric and Adolescent Medicine, Division of Pediatric Nephrology and Gastroenterology, Medical University Vienna, Vienna, Austria; 7 Medical Faculty, University Halle-Wittenberg, Halle (Saale), Germany

**Keywords:** child, joint, observer variation, omeract, rheumatic diseases, synovitis

## Abstract

**Introduction:**

Juvenile idiopathic arthritis (JIA) is the commonest rheumatologic disease in children and frequently affects the knee joint. Synovial inflammation and tenosynovitis are key pathological features, and ultrasound plays an increasingly important role in their assessment. Superb Microvascular Imaging (SMI) is a novel Doppler technique with enhanced sensitivity to low-velocity microvascular flow, but evidence on its repeatability in JIA remains limited. This study aimed to evaluate intra- and inter-observer repeatability of knee SMI in children with JIA.

**Methods:**

In this prospective multicenter study (June 2023–October 2024), 76 children with JIA were examined (Hannover Medical School and St. Josef-Stift Sendenhorst). Each underwent three standardized SMI scans: two by the same and one by a different examiner. Synovial vascularity was graded using the Pediatric OMERACT scoring system. Intra- and inter-observer reliability measures were calculated using intra-class correlation coefficients (ICC). Agreement between longitudinal and transverse suprapatellar planes was assessed using weighted kappa statistics, and correlations with clinical disease activity were analyzed via logistic regression.

**Results:**

Intra-observer reliability was excellent (ICC = 0.972, 95% CI: 0.956–0.982). Inter-observer reliability was strong (ICC = 0.828–0.928), regardless of examiner experience. Agreement between imaging planes was substantial (*κ* = 0.72, *p* = 0.32). Synovial vascularity scores correlated significantly with clinical measures of active arthritis (OR = 1.182, *p* = 0.0004), particularly with swelling (OR = 1.249, *p* < 0.0001).

**Discussion:**

SMI demonstrates excellent repeatability for assessing synovial vascularity in JIA. Its reliability, examiner independence, and non-invasive nature support its use for routine monitoring and longitudinal disease evaluation in pediatric rheumatology.

## Introduction

Juvenile idiopathic arthritis (JIA) is the most common chronic rheumatologic illness in children and adolescents, with an incidence of 2–20 per 100,000 children (1). Diagnosis remains predominantly based on clinical examination findings, with ultrasound (US) playing an increasingly important role. Among the various imaging modalities, musculoskeletal US has proven to be a reliable tool for the precise assessment of disease activity (2, 3). The knee is the most frequently affected joint and the leading cause of morbidity (1). For the diagnosis of JIA, symptoms must be present before the age of 16 years, last for at least 6 weeks, and other causes of arthritis must be excluded. Seven different subtypes exist according to the International League of the Associations for Rheumatology (ILAR), with oligoarthritis being the most prevalent (1).

Synovial inflammation and tenosynovitis are characteristic findings in JIA and can be assessed and monitored using imaging techniques that visualize synovial vascularity (SV) (4, 5). Patients with knee synovitis detected by US have a clinically meaningful risk of disease recurrence, while a substantial portion of patients in clinical remission still demonstrate active synovitis on US (6, 7). Hence, US findings could influence therapy decisions and disease course. Subclinical synovitis can only be detected by US or magnetic resonance imaging (MRI) (8–10). However, US offers advantages over MRI in terms of availability, speed, and cost.

To understand synovitis, some understanding of the related anatomical details is required. Synovial tissue lines the inner surfaces of joint capsules and bursae. It is a highly specialized and organized structure composed of two distinct histological and functional compartments: an inner avascular cellular layer adjacent to the joint or bursal cavity, referred to as the intima or lining layer, and an outer supportive vascularized layer known as the subintima or sublining layer (11). Differentiation between these two layers by ultrasound is currently not possible (11). In the acute phase of synovitis, interstitial edema and capillary hyperplasia can occur in the subintimal layer. In the subacute phase, multiple necrotizing foci form within the subintimal layer, whereas in the chronic phase, diffuse fibrosis, hypertrophic scarring, and/or keloidal tissue arise within the subintimal compartment. Part of the chronic phase includes vascular proliferation with capillary hyperplasia and telangiectasia of the subintima, with marked resetting of the entire vascular network and formation of shunt-like elements (11). This enhanced vascularity in synovitis is increasingly detectable by US in routine clinical practice.

Power Doppler ultrasound (PDUS) is the current diagnostic standard for visualizing blood flow, but it is limited in detecting small vessels with slow-velocity blood flow. Superb microvascular imaging (SMI) is a recently developed US technique designed to highlight fine, slower blood flow in microvessels without requiring contrast agents. SMI has shown a higher sensitivity than PDUS in detecting synovitis in patients with JIA due to its higher sensitivity for low-velocity microvascular flow (9, 12–16). Using advanced clutter-filter technology, SMI eliminates low-frequency motion artifacts while retaining low-speed blood signals in microvessels with a diameter of at least 250 µm at a high frame rate (17, 18). The images include a monochrome color map of blood flow, which is registered on the B-mode standard US image. Several studies have shown SMI to be superior to PDUS in detecting inflammation in various joints in patients with JIA due to its higher sensitivity for low-velocity microvascular flow (12, 15, 16, 19). In addition, SMI is as sensitive as contrast-enhanced US (20). Ünal et al. further demonstrated that periarticular vascular indices from SMI can differentiate JIA patients from healthy controls (21).

Despite promising diagnostic data, robust evidence regarding the repeatability of SMI in knee joints of JIA patients remains limited. Repeatability and reproducibility are critical to establishing credibility for SMI as diagnostic tool.

This study aims to systematically evaluate inter- and intra-observer repeatability measures of the knee using SMI in children with JIA, employing standardized scoring metrics and intra-class correlation (ICC) analyses. By quantifying reproducibility in this specific clinical context, we aim to substantiate the role of SMI as a reliable imaging modality for serial assessment and management guidance in juvenile arthritis.

## Materials and methods

### Study population

This prospective study was conducted between June 2023 and October 2024 at two different hospitals in Germany: St. Josef Hospital Sendenhorst (hospital 1) and Hannover Medical School (hospital 2). Participants were included if they were <18 years old, had a diagnosis of JIA as defined by the ILAR criteria (1), and had visited the Department of Pediatric Rheumatology of either hospital during the study period. JIA subtype and anthropomorphic data were documented. Exclusion criteria included extreme obesity, which was defined according to the inability to measure the SMI of the synovia to a maximum depth of 4 cm from skin surface without compression to allow for the standardization of measurements. Participants who could not remain still during the examination were also excluded.

Informed consent was obtained from all parents or guardians, and additionally from participants themselves if aged 12 years or older including consent for publication of human images. Ethical approval was granted by the ethics committees of the Hannover Medical School (Study registry Nr. 10852_BO_S_2023) and the Ethical Board Westfalen-Lippe (Study registry Nr. 2023-573-b-S). All procedures were carried out in accordance with relevant guidelines, regulations, and principles of the Helsinki Declaration.

### Clinical examination

All participants underwent a clinical examination of both knees within 24 h of the US examination by a board-certified pediatric rheumatologist. A dichotomous assessment (symptom present = 1, absent = 0) was applied to the presence or absence of knee pain, swelling, and reduced range of movement per knee joint. Therefore, a maximum point score of 3 points per knee was possible, and a score of ≥2 was defined as clinically active arthritis. In the hospital 1 group (*n* = 37), disease activity was additionally recorded using the validated Juvenile Arthritis Disease Activity Score (JADAS-10), as this is part of routine clinical assessment, but not in hospital 2 (*n* = 39). The JADAS-10 score was therefore only analyzed for hospital 1.

### Ultrasound examination

All US evaluations (both hospitals) were performed using a Canon Aplio i800 system using an 18 MHz linear transducer (Canon Medical Systems, Tokyo, Japan). The maximum depth of an SMI scan was 4 cm. SMI settings were 6–6.5 MHz (Doppler frequency), Color Doppler velocity of 7.2 cm/s, 0.6–1.2 kHz (Pulse Repetition Frequency), and Color Gain at 40–45, adapted to artifacts.

Both knees were examined in all participants. Examiner 1 was a board-certified pediatric radiologist with more than five years of experience in musculoskeletal ultrasound and performed all examinations consistently across both hospitals. Examiner 2 varied by site: In hospital 1, examiner 2 was a board-certified pediatric rheumatologist with >5 years of musculoskeletal US experience; in hospital 2, examiner 2 was a board-certified general pediatrician with extensive ultrasound experience but limited musculoskeletal expertise. At each hospital, examiner 2 represented a group of three physicians with the respective qualifications.

Each participant underwent a structured sequence of three ultrasound assessments. First, examiner 1 performed sonography 1. Participants then underwent an independent clinical examination by a pediatric rheumatologist, with all sonographers blinded to clinical findings. After a 1–3-h interval, participants returned for a second scan by examiner 1 (sonography 2). Immediately afterward, examiner 1 left the room, and examiner 2 performed a third examination (sonography 3).

**Figure 1:**
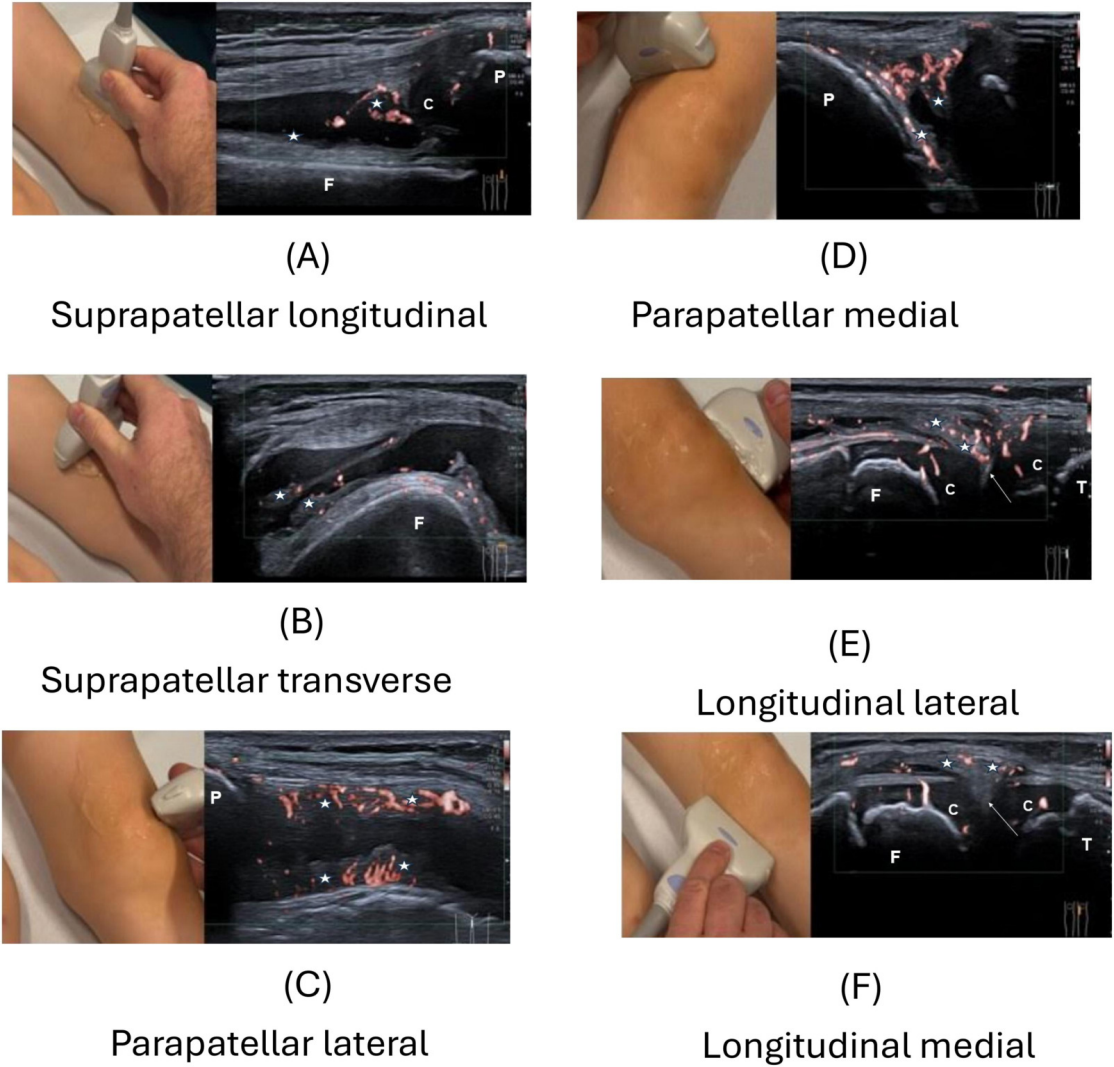
All six standard scanning positions for ultrasound (US) of the knee according to pediatric OMERACT are shown, on the right the respective US image, on the left the corresponding probe position: **(A)** suprapatellar longitudinal scan, **(B)** suprapatellar transverse scan, **(C)** parapatellar lateral transverse scan, **(D)** parapatellar medial transverse scan, **(E)** longitudinal lateral scan, and **(F)** longitudinal medial scan. C, cartilage; P, patella; F, femur; T, tibia; star, synovia; arrow, meniscus.

In accordance with previous studies (9, 22, 23), six standard scanning positions of the knee were included: the suprapatellar recess in longitudinal and transverse, the lateral and medial parapatellar scans, and the longitudinal medial and lateral joint-line scans covering the meniscal region (Figure 1, Supplementary Video Clip S1, S2 as Supplementary Material) (9, 23, 24). These positions encompassed all five recommended by the Pediatric Rheumatology European Society (PReS) Imaging Working Party (9). Before each examination, participants flexed and extended their knee three times (23). For the suprapatellar recess, examination of the knee in 20–30° flexion is recommended as SV detection rates are higher than in neutral position (9). However, this is only proven for the suprapatellar recess and Ricci et al. advocate US in a neutral position of the joint to avoid unintentional stretching/compression of small vascular elements, which might collapse and disappear during Doppler imaging (11). Due to these arguments and because 30° flexion was not part of the routine protocol at either center, all examinations were performed in a neutral relaxed position. In addition, the aim of our study was not maximum detection rate of SV but intra- and inter-observer repeatability in a routine setting.

For statistical analysis, the knee with the higher vascularity score on sonography 1 was selected. However, if scores were identical bilaterally, the right knee was analyzed. Between one and eight participants were examined per day, and the order of sonography 2 was randomized to minimize recall bias for examiner 1. Synovial vascularity (SV) was graded during each examination, immediately recorded, and transferred to a study nurse. Scores from all six scans were summed to yield a total SV score. All data were pseudonymized.

### Scoring of synovitis

**Figure 2:**
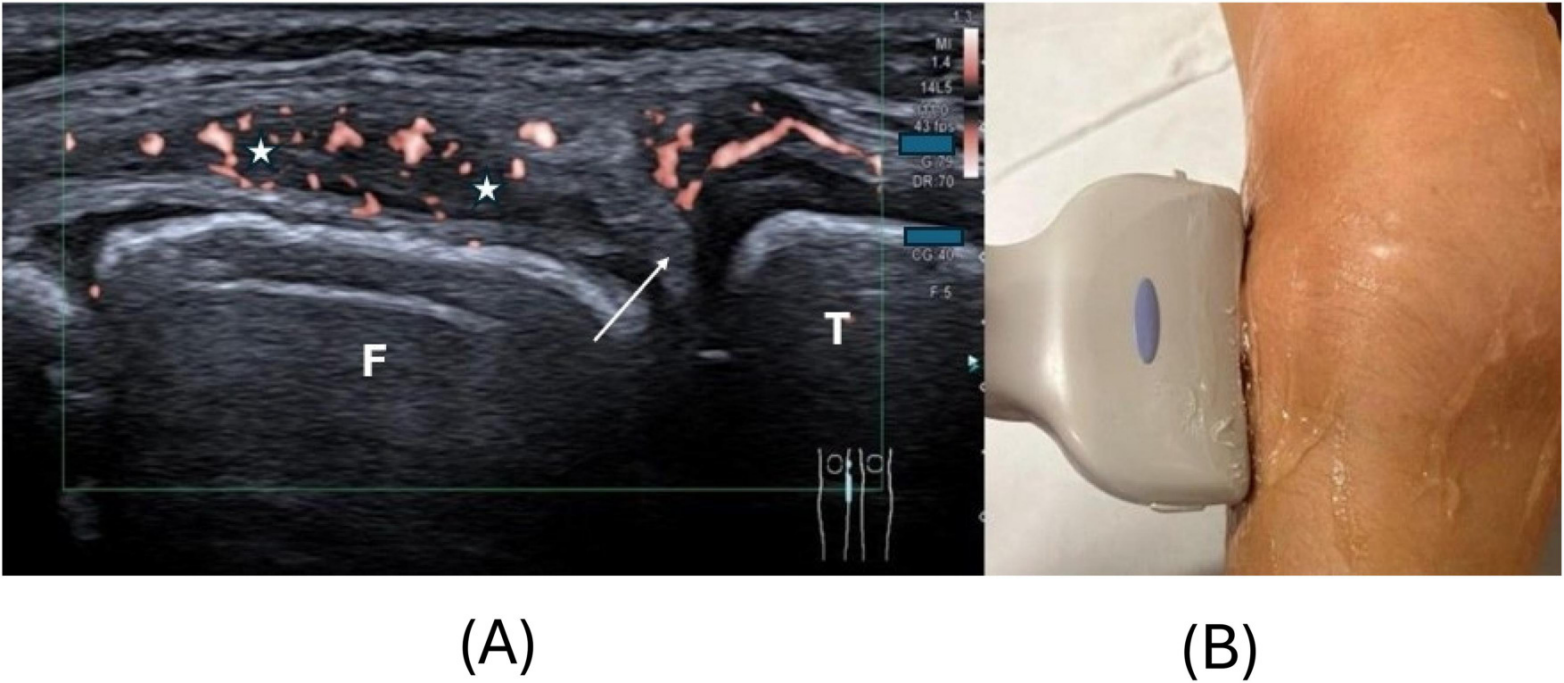
**(A)** Shows a lateral longitudinal scan of the knee in a 9-year-old female patient with juvenile idiopathic arthritis and intra-synovial hypervascularity (orange signals) OMERACT (Outcome Measures in Rheumatology) grade 2. The synovial tissue (star) is hypoechoic and thickened with vascular signals in less than 30% of the synovial tissue. Positioning of the probe slightly anterior to the iliotibial band is demonstrated in **(B)**, the arrow shows the meniscus as hyperechoic triangle. F, distal femur, T, proximal tibia.

**Figure 3:**
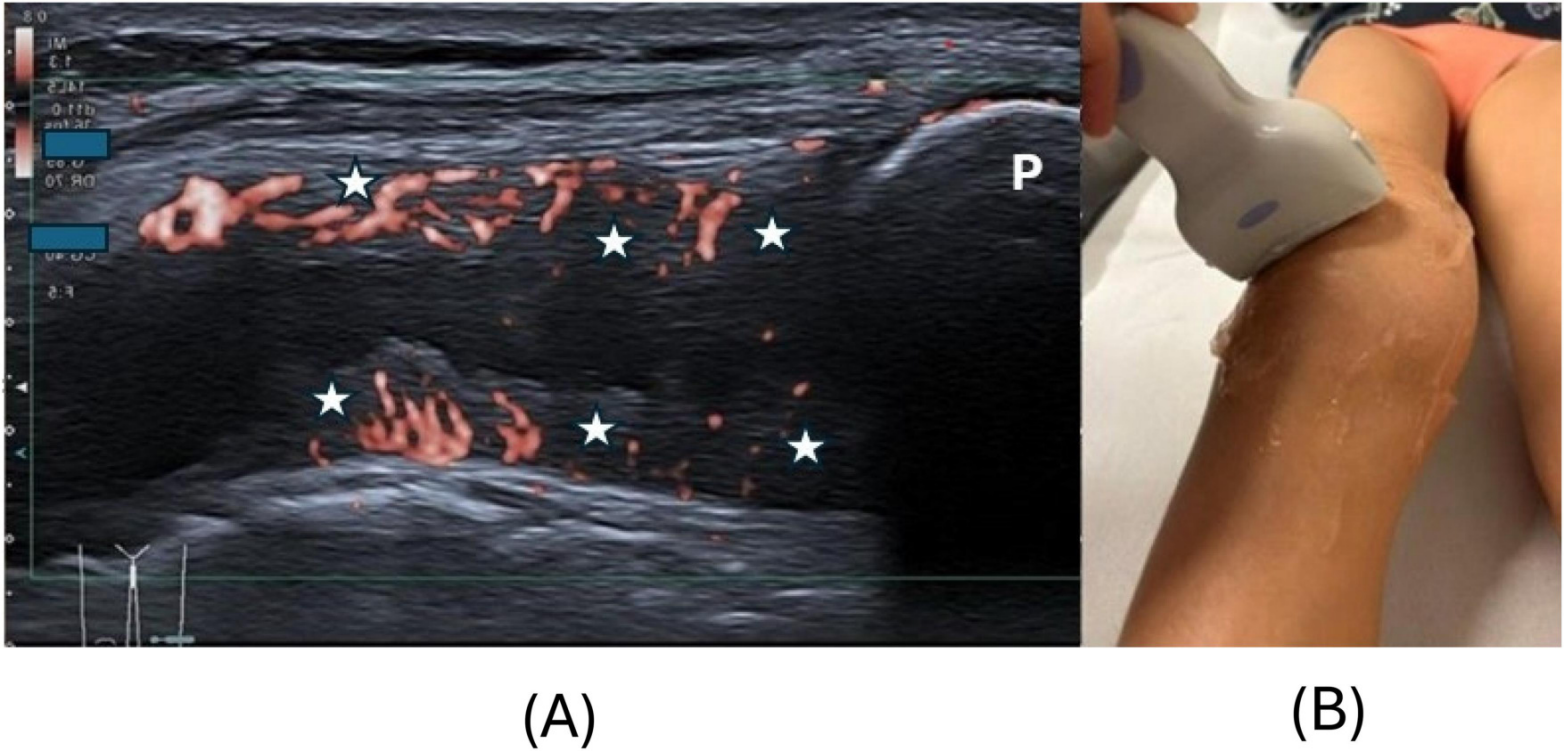
**(A)** Shows a lateral parapatellar scan of the knee in a 7-year-old female patient with juvenile idiopathic arthritis and with intra-synovial hypervascularity [**(A)**, orange signals] OMERACT (Outcome Measures in Rheumatology) grade 3. The synovial tissue (star) is hypoechoic and thickened with intense hypervascularity covering more than 30% of the synovial tissue. Note that in some cases, the tissue overlying the distal femur may correspond to hypertrophic synovial tissue but also to the prefemoral fat pad. This is an anatomical pitfall. Positioning of the probe is demonstrated in **(B)**. P, patella.

Synovitis on US was defined as abnormal intra-articular, anechoic or hypoechoic, and non-displaceable material with color Doppler signals detected within synovial hypertrophy (4). The probe was gently positioned, and ultrasound gel was applied generously during measurements to avoid mechanical interference with synovial microvascularity. Synovial hypertrophy was defined, according to the Outcome Measures in Rheumatology (OMERACT) Clinical Trials Definitions Ultrasound Task Force, as non-displaceable, poorly compressible, abnormal hypoechoic or relative to the subdermal fat isoechoic thickened intra-articular tissue lining the recess (25). Scoring of SV was carried out according to the Pediatric OMERACT scoring system: Normal Doppler (grade 0) described the complete absence of signal; grade 1 = few individual dots of synovial Doppler signals; grade 2 = confluent Doppler signals, but representing less than 30% of the visible synovial tissue (Figure 2); and grade 3 = confluent Doppler signals in more than 30% of the visible synovial tissue (26) (Figure 3). The area in relation to which this percentage is determined was restricted to the synovial tissue only and not to the entire joint (and possibly effusion) visualized in the image, as this might have resulted in a lower grade. In the lateral parapatellar transverse position, “deep” vascular signals overlying the femur may in some cases be related to hypertrophic synovial tissue but also to the prefemoral fat pad (Figure 3) (27). This anatomical pitfall received particular attention from all examiners. Feeding vessels, defined as single pulsating vessels clearly larger than the net-like appearance of the microvasculature of the synovia, were not counted as increased SV, as they are a normal finding in developing children (4).

### Comparison of longitudinal vs. transverse scanning positions

Agreement between longitudinal and transverse imaging planes was assessed at the suprapatellar recess and calculated separately for sonography 1 and 3, and for hospitals 1 and 2. For lateral and medial parapatellar views, comparisons with longitudinal planes were not possible as the positions differ. The parapatellar scanning positions are located higher and at the level of the patella, whereas the longitudinal positions are positioned below the patella at the level of the meniscus.

### Statistical analysis

Statistical analyses were performed using SAS (SAS Institute, Cary, NC, USA, 9.4) and R (R Core Team 2024, 4.4.1, R Foundation for Statistical Computing, Vienna, Austria). A two-way random-effects model was used to calculate intra-observer reliability. Sample size was determined based on the result that 71 patients were needed to achieve a power of 90% to demonstrate that the entire 95% confidence interval for an ICC is above 0.7, when the true ICC is 0.85. Comparison between longitudinal and transverse orientation for suprapatellar recess was calculated using the McNemar–Bowker test for symmetry, and weighted kappa coefficients were determined. Relationships between imaging and clinical examination were assessed using logistic regression. Data are presented as mean ± SD, unless otherwise stated. Significant inter-observer reliability was calculated using a one-way random-effects model. A significance level of 5% was used, and 95% confidence intervals were computed where appropriate.

## Results

### Study population

A total of 76 pediatric participants [58 females, age mean 9.5 years (SD 4.325]), each with a diagnosis of JIA, were included. Participant characteristics are summarized in Table 1. The participant cohort from hospital 1 comprised 37 participants [29 females, age mean 9.1 years (SD 4.26)], and hospital 2 included 39 participants [27 females, age mean 9.9 years (SD 4.40)]. Comparison of age between the cohorts of hospital 1 and hospital 2 showed no significant differences by *t*-test (*p* = 0.385) or Wilcoxon test (*p* = 0.332). In 34 participants, SV was higher in the right knee and in 17 participants in the left knee. In 25 of the 76 participants, both knees showed identical SV scores, after which the right knee was chosen for evaluation. Complete inter-observer data were available for 74 children due to missing second-reader assessment in two participants from hospital 1. In one participant from hospital 2, the clinical assessment was missing. Therefore, 73 patient datasets were used for correlation of clinical symptoms and SV score. No participants were excluded due to extreme obesity or excessive motion during US.

**Table 1:** Participant characteristics.

Participant characteristics	All participants	Hospital 1 (experienced)	Hospital 2 (unexperienced)
Number of participants	76	37	39
Gender distribution male/female	20/56	8/29	12/27
Age, median (IQR)	10 (6;13)	9 (6;12)	10 (6.75;14)
Age, mean (SD)	9.5 (4.33)	9.1 (4.26)	9.9 (4.40)
persistent oligoarthritis, *n*	40	12	28
Extended oligoarthritis, *n*	12	8	4
Seronegative polyarthritis, *n*	7	4	3
Oligoarthritis, *n*	6	6	0
Psoriasis arthritis	5	3	2
Enthesitis-associated arthritis, *n*	4	2	2
Undifferentiated arthritis, *n*	2	2	0
Clinically active arthritis, *n*	36	23	13
Clinical arthritis score, mean (SD)	1.52 (1.2)	1.68 (1.29)	1.37 (1.1)
JADAS-10, mean (SD)	n. a.	12.9 (18.0)	n.a.

IQR, interquartile range 1;3; JADAS, Juvenile Arthritis Disease Activity Score; *n*, number of participants; n. a., data not available; SD, standard deviation; SV, synovial vascularity.

### Intra-observer reliability

The resulting ICC was 0.972 (95% CI: 0.956–0.982), indicating excellent repeatability. This value was significantly greater than 0.9 (*p* < 0.0001), confirming high intra-observer reliability in SMI assessment.

### Inter-observer reliability

Using a one-way random-effects model, comparison of sonography 1 vs. sonography 3 for the entire cohort revealed an ICC of 0.888 (95% CI: 0.828–0.928). Comparison of sonography 2 vs. sonography 3 revealed an ICC of 0.867 (95% CI: 0.798–0.914) (Table 2).

**Table 2:** Inter-observer reliability of Outcome Measures in Rheumatology (OMERACT) score to assess synovial vascularity.

Sonography	ICC (CI) of sonography 1 vs. sonography 3
Sonography 3 all participants *n* = 74	0.888 (95% CI: 0.828–0.928)
Sonography 3 hospital 1 *n* = 35	0.923 (95% CI: 0.854–0.960)
Sonography 3 hospital 2 *n* = 39	0.856 (95% CI: 0.744–0.922)

CI, confidence interval; ICC, intra-class correlation coefficient; *n*, number of participants; SD, standard deviation.

When analyzed by recruitment center, strong agreement across readers was found. Inter-observer correlation between sonography 1 and sonography 3 was slightly higher in hospital 1, where the second examiner had extensive experience in musculoskeletal US, compared to hospital 2, where the second examiner had limited experience (Table 2).

### Association between imaging and clinical examination

Association analysis between SV scores and clinical scores was performed in 75 participants, of whom 35 met the criteria for active JIA (clinical score ≥2) (Table 3). The association between the total SV score (sum of six sub-scores from sonography 1) and clinical classification (“active JIA” based on ≥2 of joint pain, restricted motion, or swelling) was evaluated using logistic regression (Table 3). Participants with clinically active arthritis demonstrated higher SV scores than those without clinical signs (Table 4); however, SV scores in individuals with clinical score 3 were lower than in those with score 2 (Table 4). The highest SV scores consistently appeared in clinical score 2 and in the lateral parapatellar position. In clinical score 3, both suprapatellar positions yielded the lowest SV scores, whereas both parapatellar positions produced the highest. Three clinically normal joints (score 0) showed SV grades 2–3, suggesting subclinical arthritis, while six clinically active cases (score ≥2) showed no hypervascularity. In addition, ten participants (mostly from hospital 2) exhibited clinical score 1 but elevated SV scores (grade 2–3). When counting the highest single SV subscore, 36% (5/14) of participants with a clinical score of 2 had an SV score of 3, whereas 48% (10/21) of participants with a clinical score of 3 also showed an SV score of 3.

**Table 3:** Association between clinical signs of arthritis and the superb microvascular imaging synovial vascularity score.

Clinical arthritis score	Synovial vascularity score		
	*p*	Odds ratio	*n* (%)
Clinically active inflammation^a^	0.0008	1.173 (95% CI: 1.068–1.288)	35 of 75 (47%)
Knee pain (yes/no)	0.0205	1.106 (95% CI: 1.016–1.205)	34 of 75 (45%)
Restriction of motion (yes/no)	0.0021	1.152 (95% CI: 1.053–1.261)	33 of 75 (44%)
Swelling (yes/no)	< 0.0001	1.241 (95% CI: 1.114–1.383)	39 of 75 (52%)

*n*, number of participants; CI, confidence interval.

a Defined as 2 points or more in the clinical arthritis score.

**Table 4:** OMERACT scores in different probe positions in relation to clinical arthritis score.

Scanning position and OMERACT score in sonography 1 (mean ± SD)	Clinical score 0 *n* = 25	Clinical score 1 *n* = 15	Clinical score 2 *n* = 14	Clinical score 3 *n* = 21
Sum of all six scanning positions	1.20 ± 3.30	6.80 ± 5.35	8.14 ± 5.67	7.76 ± 5.51
Suprapatellar longitudinal	0.20 ± 0.58	0.87 ± 0.92	1.21 ± 1.12	0.95 ± 1.07
Suprapatellar transverse	0.20 ± 0.71	1.27 ± 1.03	1.29 ± 1.33	1.19 ± 1.08
Lateral longitudinal	0.20 ± 0.50	1.20 ± 1.15	1.50 ± 1.09	1.48 ± 1.23
Lateral parapatellar	0.32 ± 0.75	1.47 ± 0.99	1.50 ± 1.16	1.48 ± 1.03
Medial longitudinal	0.12 ± 0.44	0.67 ± 0.98	1.29 ± 0.99	1.24 ± 1.14
Medial parapatellar	0.16 ± 0.62	1.33 ± 1.23	1.36 ± 1.22	1.43 ± 1.16

OMERACT, Outcome Measures in Rheumatology; SD, standard deviation; *n*, number of participants.

### Comparison of scanning positions

Agreement between SV in the longitudinal and transverse imaging planes of the suprapatellar recess showed no differences across transverse and longitudinal orientations, test centers, or sonographers. For sonography 1, all 76 participants were included, and weighted kappa in Mc Nemar–Bowker test was 0.7021 (95% CI: 0.5917–0.8124), demonstrating substantial agreement and revealing no significant deviation from symmetry, i.e., no deviation between proportions (*p* = 0.3173). Due to a missing value, 75 participants were included for sonography 3 and weighted kappa in the Mc Nemar–Bowker test was 0.7462 (95% CI: 0.6270–0.8654), again demonstrating substantial agreement without significant deviation from symmetry (*p* = 0.3720). When considering hospitals 1 and 2 separately, the results were as follows: In hospital 1, sonography 1 (*n* = 37) had a weighted kappa of 0.7113 (95% CI: 0.5540–0.8686), *p* = 0.8494, and sonography 3 (*n* = 36) had a weighted kappa of 0.6170 (95% CI: 0.4060–0.8281), *p* = 0.8647. In hospital 2, sonography 1 (*n* = 39) had a weighted kappa of 0.6925 (95% CI: 0.5406–0.8444), *p* = 0.2147, and sonography 3 (*n* = 39) showed a weighted kappa of 0.8532 (95% CI: 0.7423–0.9641), *p* = 0.4232.

In hospital 1, the highest SV score was measured in both lateral scanning positions by both examiners. In hospital 2, the parapatellar scanning positions showed the highest SV for examiner 1, but for examiner 2, the suprapatellar transverse and parapatellar lateral scanning positions revealed the highest SV score (Table 5).

**Table 5:** OMERACT scores of synovial vascularity in relation to probe position and hospital.

Scanning position	OMERACT score of SV mean (± SD)					
	Sonography 1			Sonography 3		
	All P	Hospital 1 participants	Hospital 2 participants	All P	Hospital 1 participants	Hospital 2 participants
Knee score (sum of all positions)	5.38 ± 5.67	5.92 ± 6.06	4.87 ± 5.30	4.66 ± 5.26	4.49 ± 5.05	4.82 ± 5.50
Suprapatellar longitudinal	0.72 ± 0.97	0.84 ± 1.07	0.62 ± 0.88	0.73 ± 0.96	0.72 ± 0.94	0.74 ± 0.99
Suprapatellar transverse	0.88 ± 1.11	0.89 ± 1.15	0.87 ± 1.08	0.80 ± 1.00	0.69 ± 0.95	0.90 ± 1.05
Lateral longitudinal	0.99 ± 1.10	1.19 ± 1.22	0.79 ± 0.95	0.78 ± 1.01	0.80 ± 1.02	0.77 ± 1.01
Lateral parapatellar	1.08 ± 1.09	1.08 ± 1.16	1.08 ± 1.04	0.85 ± 1.06	0.89 ± 1.11	0.82 ± 1.02
Medial longitudinal	0.75 ± 1.01	0.97 ± 1.07	0.54 ± 0.91	0.70 ± 1.03	0.60 ± 0.98	0.79 ± 1.08
Medial parapatellar	0.96 ± 1.17	0.95 ± 1.15	0.97 ± 1.20	0.77 ± 1.05	0.75 ± 1.08	0.79 ± 1.03

OMERACT, Outcome Measures in Rheumatology; P, participants; SV, synovial vascularity; SD, standard deviation.

An infographic summary of this study is provided in Supplementary Material S3.

## Discussion

This study demonstrates that SMI of the knee in children with JIA is highly repeatable and correlates significantly with clinical disease activity. Using both intra- and inter-observer assessments, excellent reliability in synovial vascularity grading was confirmed, supporting the utility of SMI as a consistent and examiner-independent imaging modality for monitoring synovitis of the knee in participants with JIA.

### Repeatability of SMI

The intra-observer intra-class correlation coefficient (ICC) of 0.972 (95% CI: 0.956–0.982) confirms near-perfect reproducibility when the same rater evaluates the same joints across two time points. This aligns with other musculoskeletal US studies with similarly high ICC values (28–30).

Inter-observer ICCs, ranging from 0.856 to 0.923 across different comparisons and centers, also indicate strong agreement between readers. While slightly lower than intra-observer values, these results reflect perfectly acceptable variability in real-world multi-reader settings. Interestingly, inter-observer reliability was higher in the hospital 1 subgroup with sonographers experienced in musculoskeletal US, possibly reflecting site-related consistency in acquisition protocols or reader training.

### Comparison of SMI scores in different imaging positions

No significant differences were observed between longitudinal and transverse scans in the suprapatellar region with weighted kappa values showing substantial agreement (*κ* = 0.70). These findings suggest that both orientations are largely interchangeable for suprapatellar assessment, although a higher detection rate of SV could be expected in the transverse orientation as the entire recess is examined. Windschall et al. found SV to be highest in the suprapatellar transverse, the two parapatellar, and the lateral longitudinal scans in JIA (9). However, this was not observed in our cohort, possibly because the difference between longitudinal and transverse probe orientation in the suprapatellar recess not large enough to result in a higher grading in the OMERACT score.

In this study, the highest SV scores were found in the lateral and parapatellar scanning positions, underscoring the need to examine all six positions with particular attention to parapatellar scans.

### Clinical relevance of SMI findings

This study demonstrates a strong association between SMI-derived SV scores and clinical assessment of knee inflammation. The summed SMI score significantly predicted clinical inflammation status (OR 1.173, *p* = 0.0008), with an even stronger association for swelling alone (OR 1.241, *p* < 0.0001) and a weaker association for patient-reported pain (OR 1.06, *p* = 0.0205). Unexpectedly, SV scores across most scanning positions were slightly higher in participants with a clinical score of 2 than in those with a score of 3, which may hypothetically reflect inflammation in grade 3 cases being more focal, or the presence of chronic fibrotic synovial changes associated with reduced hypervascularity (11). To test repeatability, all six scanning positions were scored and summed in this study. However, in a clinical setting, the highest score is usually taken, even if only encountered in one scanning position. Accordingly, when counting single highest and not summed SV scores, 48% of participants with a clinical score of 3 also had an SV score of 3, as compared to participants with a clinical score of 2 who had an SV score of 3 in only 36%.

Three clinically normal joints demonstrated increased SV (grade 2–3), suggesting subclinical synovitis, consistent with previous reports (6, 7). Moreover, ten participants with a clinical score of 1—found in 8/10 cases presenting with swelling—exhibited high SV scores, suggesting that swelling may represent the most sensitive clinical indicator of active arthritis.

These observations align with studies showing that subclinical synovitis can be detected using PDUS (6, 7). Given the higher sensitivity of SMI (13), broader clinical use may increase detection of early or subclinical disease. This study also demonstrates excellent intra- and inter-observer reproducibility, confirming that SMI reliably measures SV in JIA, independent of operator experience. While previous work has shown SMI to be superior to PDUS in detecting synovial vascularity (13), this is the first study to provide comprehensive proof of method and reproducibility across different clinical settings.

Although MRI remains the reference standard for detecting knee arthritis in JIA (8, 10, 22), recent data indicate high diagnostic accuracy of knee US compared with MRI (24). Combined with SMI's sensitivity and demonstrated reproducibility, these findings support its potential as a timely, accessible, and cost-effective tool for assessing synovitis in JIA.

### Strengths and limitations

A major strength of this study is the robust methodological approach, including the use of ICCs based on random-effects models, as recommended by biometricians, and the multicenter design, which enhances generalizability. In addition, physicians with both very low and very high levels of experience in musculoskeletal US were included, allowing assessment of the possible influence of physician experience on repeatability of SMI. Hence, the study demonstrated that even physicians with limited experience in musculoskeletal US achieved good inter-observer repeatability. This confirms SMI as a robust and examiner-independent method. Furthermore, the sample size (*n* = 76) is substantial for a pediatric imaging reliability study.

Several limitations should be acknowledged. Although obesity was not an exclusion criterion, SMI measurements were limited to a maximum depth of 4 cm. While studies in solid organs have demonstrated SMI imaging at depths up to 6 cm (31), this study did not assess performance in cases of extreme obesity, where synovitis detection may be less reliable. Therefore, detection of arthritis in extreme obesity remains a technical uncertainty, as also noted by Ricci et al. (11). Another minor limitation is the limited assessment of inter-day variability, since intra-observer repeatability was tested within hours. However, we believe this to be methodologically sufficient for the aims of this study.

Finally, while SMI vascularity grades correlated with clinical disease activity, this analysis was performed to test reliability of the investigation. Future work could focus on the histological evaluation of the SMI findings compared to clinical findings, possibly requiring additional validation against MRI findings, which was beyond the scope of this study.

The current OMERACT scoring system used in this study was based on the percentage of hypervascularized synovial tissue using PDUS and not SMI, even though SMI was used in this study (26). As SMI detects more blood vessels (Supplementary video clip S2) than PDUS (Supplementary video clip S3), this possibly leads to a higher score of synovitis and disease activity, which should be taken into consideration when using SMI and OMERACT together. Therefore, the current OMERACT scoring system may need to be revalidated for the use with SMI.

## Conclusions and outlook

In summary, these findings support the excellent intra- and inter-observer repeatability of SMI for detecting synovial vascularity in the knee of children with JIA. The technique proved reliable and robust across different synovitis grades and levels of physician experience, and demonstrated significant clinical relevance, reinforcing its role as a valuable tool for both acute and longitudinal assessment of synovitis. This study provides further evidence for the use of SMI as a predictive marker of disease progression and treatment response. Studies correlating SMI with MRI might further establish SMI as a reliable alternative, given its substantially lower cost, shorter examination time, and reduced logistical burden compared to MRI examinations in children with JIA.

## Data Availability

The raw data supporting the conclusions of this article will be made available by the authors, without undue reservation.

## Supplementary material

Supplementary Video Clip S1 This short video clip demonstrates conventional power Doppler ultrasound (PDUS) of the knee in a 7-year-old female patient with juvenile idiopathic arthritis. The synovia shows marked hypoechoic hypertrophy. Hypervascularity visualized by PDUS is moderate. Note the hypoechoic joint effusion in the joint recess showing no vascularization.

Supplementary Video Clip S2 This short video clip demonstrates synovial hypertrophy and hypervascularity visualized with superb microvascular imaging (SMI) ultrasound in the same patient as shown in S1. In comparison to Power Doppler US, SMI reveals greater intrasynovial hypervascularity, visualized as a fine microvascular network.

Supplementary Infographics S3 Infographic summary of this study.
