# Supplementary figures and images for: Superb microvascular imaging ultrasound of the knee in patients with juvenile idiopathic arthritis—a repeatability study
Source: Front Pediatr. 2026 Jan 26;13:1759370. doi: 10.3389/fped.2025.1759370 (PMC12883735; doi:10.3389/fped.2025.1759370)

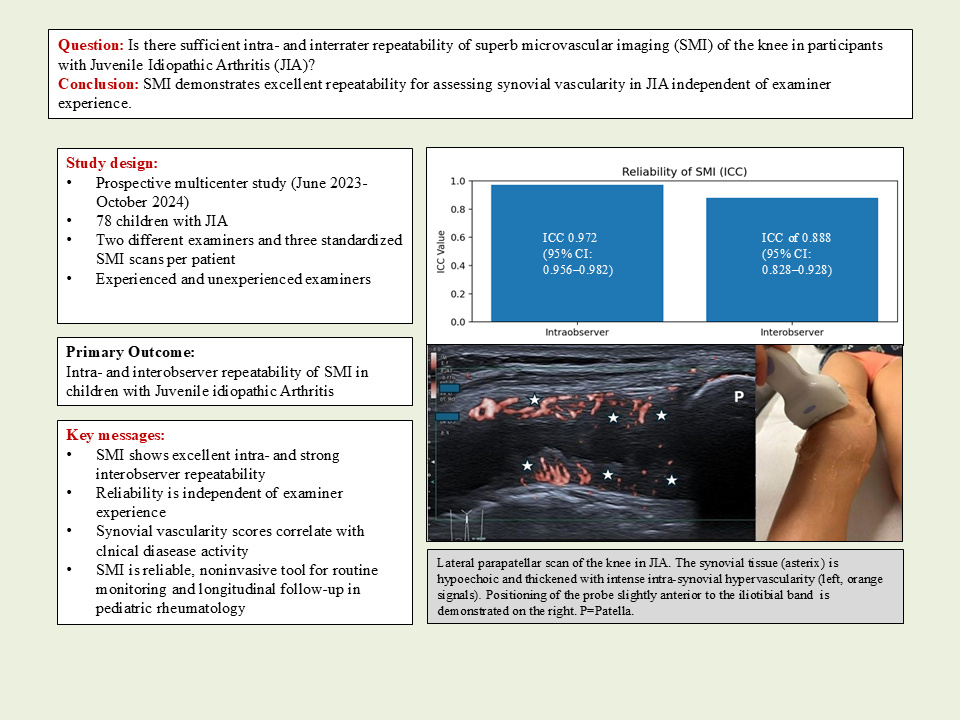

Supplement: Supplementary Infographics S3 — Infographic summary of this study. [file Image1.jpeg]
